# Dicer-2-Dependent Activation of *Culex* Vago Occurs via the TRAF-Rel2 Signaling Pathway

**DOI:** 10.1371/journal.pntd.0002823

**Published:** 2014-04-24

**Authors:** Prasad N. Paradkar, Jean-Bernard Duchemin, Rhonda Voysey, Peter J. Walker

**Affiliations:** 1 CSIRO Animal, Food and Health Sciences, Australian Animal Health Laboratory, Geelong, Victoria, Australia; 2 CSIRO Ecosystem Sciences, Australian Animal Health Laboratory, Geelong, Victoria, Australia; Colorado State University, United States of America

## Abstract

Despite their importance as vectors of human and livestock diseases, relatively little is known about innate antiviral immune pathways in mosquitoes and other insects. Previous work has shown that *Culex* Vago (*Cx*Vago), which is induced and secreted from West Nile virus (WNV)-infected mosquito cells, acts as a functional homolog of interferon, by activating Jak-STAT pathway and limiting virus replication in neighbouring cells. Here we describe the Dicer-2-dependent pathway leading to WNV-induced *Cx*Vago activation. Using a luciferase reporter assay, we show that a NF-κB-like binding site in *Cx*Vago promoter region is conserved in mosquito species and is responsible for induction of *Cx*Vago expression following WNV infection. Using dsRNA-based gene knockdown, we show that the NF-κB ortholog, Rel2, plays significant role in the signaling pathway that activates *Cx*Vago in mosquito cells *in vitro* and *in vivo*. Using similar approaches, we also show that TRAF, but not TRAF-3, is involved in activation of Rel2 after viral infection. Overall the study shows that a conserved signaling pathway, which is similar to mammalian interferon activation pathway, is responsible for the induction and antiviral activity of *Cx*Vago.

## Author Summary

Viruses like West Nile, dengue and Japanese encephalitis are responsible for large number of human and livestock diseases worldwide. These viruses, transmitted by female mosquitoes via saliva during blood-feeding, elicit an immune response in these mosquitoes. The details of this immune response are still being investigated. Dicer2, which has previously been shown to be involved in RNAi mediated antiviral activity in mosquitoes, contains helicase domain, which leads to activation of antiviral protein, Vago. Vago is functionally similar to mammalian interferon and after secretion activates Jak-STAT pathway in neighboring cells leading to antiviral effect. Here we demonstrate that sensing of viral RNA by Dicer2 leads to activation of TNF receptor-associated factor (TRAF), which in turn leads to cleavage and release of amino-terminal domain of Rel2, NF-κB ortholog. Rel2 binds to a conserved NF-κB binding site on Vago promoter region leading to its induction. This proposed mechanism of Vago activation is similar to mammalian interferon activation after viral infection. The identification of this novel and evolutionarily conserved pathway downstream of Dicer2 provides new insight into the immune signalling in mosquitoes and other invertebrates.

## Introduction

Hematophagous insects (mosquitoes, sand flies and midges) serve as vectors of many important viral diseases of humans and livestock. Arthropod-borne viruses are endemic or seasonally epidemic through most regions of the world, accounting for inestimable numbers of human infections, significant rates of mortality, and major impacts on livestock production and trade [1], [2]. Indeed it has been estimated that over 25% of all emerging viral diseases globally are vector-borne [3] with factors such as global warming, increased population densities and ease of world travel driving the spread into new geographical areas [4], [5].

Despite their importance in the transmission of viral disease, knowledge of the processes of infection and immunity in insects (and other invertebrates) is relatively poor. Insects are known to lack key components of vertebrate immune system, such as interferons, antibodies, lymphocytes and other elements of innate and adaptive immunity [6]. RNA interference (RNAi) has been shown to be a major aspect of the insect antiviral response to a wide range of both RNA viruses and DNA viruses [7], [8]. This involves the detection and degradation of viral double-stranded (ds) RNA by Dicer-2, generating short interfering (si) RNAs (siRNA). These viral-siRNAs are subsequently incorporated into the RNA-induced silencing complex (RISC) and used to guide the targeted degradation of viral RNAs [9]. There is also evidence that some evolutionarily conserved immune signaling pathways, such as Toll receptor [10], Imd [11] and Jak-STAT [12], which direct the induction of antimicrobial peptides in *Drosophila*, are also involved in the antiviral response. The Toll pathway was initially identified using a *Drosophila* mutant screen to be involved in the antiviral response [13], and subsequently was shown to have a role in limiting dengue virus infection in *Aedes aegypti* mosquitoes [10]. In contrast, using a Sindbis virus replicon system, it has been reported that *Drosophila* mutants deficient in Toll pathway transcription factors had no effect on viral replication, but that replication was enhanced in mutants deficient in the elements of the Imd pathway [11]. The Jak-STAT pathway was also initially found to be important in antiviral signalling in *Drosophila* [12] and later has been shown to be play significant role in immunity to dengue virus in mosquitoes [14].

Recently, it has also been shown that Dicer-2, which is central to the RNAi response, also mediates signaling that leads to induction of Vago, a secreted peptide that also has a role in antiviral immunity in *Drosophila* and mosquitoes [15], [16]. Through a mechanism that is independent of the RNAi pathway, it has been shown in mosquito cells that dsRNA-induced, Dicer-2-mediated secretion of Vago inhibits West Nile virus (WNV) infection by activating Jak-STAT pathway in the neighboring cells [15]. This suggests that, although structurally unrelated, Culex Vago may have a function similar to mammalian interferons [15].

In vertebrates, viral infection leads to recognition of viral dsRNA by DExD/H-box helicases like RIG-I or MDA-5 [17]. These viral sensors interact with mitochondrial antiviral signaling (MAVS) protein via CARD domain [18], leading to activation of TNF receptor-associated factors (TRAF)-3/6 [19]. This is followed by activation and nuclear localization of interferon regulatory factors (IRFs)-3/7 and/or nuclear factor-kappa B (NF-κB). NF-κB activation involves phosphorylation and degradation of inhibitory kappa B (IκB) which releases NF-κB to translocate into the nucleus [20]. The IRFs and NF-κB by bind to the promoter region of the gene and induce interferon beta via TRAF3 and TRAF6 respectively [21]–[23]. In mosquitoes and other invertebrates viral dsRNA is recognized by Dicer-2, which contains an amino-terminal DExD/H-box helicase domain similar to RIG-I, ultimately leading to induction of Vago [15], [16]. However, the intermediate proteins involved in this activation have not been identified.

In this report, we investigate the Vago activation pathway following flavivirus infection of mosquito cells *in vitro* and *in vivo*. We show that induction of Vago by West Nile virus (WNV) or dengue virus (DENV) is mediated by Dicer-2-dependent activation of an ortholog of TRAF, which in turn activates the NF-κB ortholog, Rel2. We also demonstrate that Vago activation is dependent on a conserved NF-κB binding site in the upstream promoter region, suggesting that flavivirus-induced Vago activation occurs by an evolutionarily conserved mechanism.

## Materials and Methods

### Cell culture maintenance and viral infection

Hsu (*Culex quinquefasciatus*) and RML-12 (*Aedes albopictus*) cells were maintained at 28°C in Leibovitz's L-15 medium (Gibco #11415) containing 10% tryptose phosphate broth solution, 15% heat-inactivated fetal bovine serum, and 1% penicillin-streptomycin solution. West Nile virus (NY99 and Kunjin strains) and dengue-2 virus (NGC strain) were used for the study. C6/36 (*Aedes albopictus*) cells were maintained in RPMI media at 28°C were used to propagate the virus. Vero cells maintained in EMEM at 37°C were used for plaque assays.

### Luciferase reporter construction and assay

Luciferase reporter constructs were prepared by cloning the 2007 bp region upstream of the initiation codon of the *Culex* Vago gene. For the core promoter, the region from −10 to +20 bp (in relation to the transcription start site) was used. PCR was performed on *Culex* genomic DNA to amplify the region (DS231818; region 720994–723000), and using restriction digests to clone it upstream of *Renilla* luciferase reporter gene. A similar strategy was used to clone sequential deletions of the promoter region in the same vector. Mutations were introduced into the Rel binding site sequence (CTT→AGG) using QuickChange Site-Directed Mutagenesis kit (Stratagene) according to the manufacturer's protocol. The plasmid was transfected into Hsu cells using Cellfectin (Invitrogen) in serum-free medium along with plasmid containing firefly luciferase reporter gene driven by the insect cell promoter *OpIE*2. The luciferase reporter assay was performed on cell lysates using Dual-Luciferase Reporter Assay System (Promega) according to the manufacturer's instructions. The experiments were conducted at least 3 times, each in triplicates. The controls were set arbitrarily at 1, and fold-increase over control was plotted as mean ± SD.

### RNA extraction and real-time RT-qPCR

Total RNA was extracted from cells using the Qiagen RNA extraction kit according to the manufacturer's protocol. Reverse transcription was performed by random hexamers using the First Strand Synthesis kit (Invitrogen). Real-time RT-qPCR was performed using gene-specific primers (Supplementary Table S2). As an internal control, real-time RT-qPCR was also performed using the housekeeping gene, RpL32. The control was set arbitrarily at 1 and fold-increase over control was calculated by the ΔΔCt method. The experiments were conducted at least 3 times, each in triplicates. The results were plotted in graph format as mean ± SD.

### Cell lysis and western blotting

Cells were lysed in RIPA lysis buffer containing protease inhibitor. The cell lysates were collected by centrifuging at 16,000×*g* for 10 min at 4°C. Protein samples (10 µg) were loaded onto polyacrylamide gels (4–12%). After electrophoresis and transfer to nitrocellulose membranes, proteins were blotted using anti-Vago [15] or anti-V5 (Invitrogen) antibody followed by anti-rabbit or anti-mouse secondary antibody, respectively. After adding substrate, the membrane was exposed to film to detect protein levels. Anti-beta-actin antibody was used in immunoblots as a loading control.

### Plaque assays

Plaque assays were performed as previously described [15]. In brief, the supernatant media from the cells infected with WNV (10-fold dilutions) were added onto confluent Vero cell monolayers in 6-well plates. After 1 h incubation at 37°C, the cells were overlaid with medium containing agar. Plaques formed within 72 hpi were counted and the results were plotted graphically. The experiments were conducted at least twice, each with duplicates.

### dsRNA preparation and transfection

Gene-specific dsRNA (∼400 nt) were prepared using the MEGAscript RNAi kit according to the manufacturer's protocol. dsRNAs were transfected into Hsu cells using Cellfectin according to a previously described protocol [15]. dsRNA against green fluorescent protein (GFP) was used as a specificity control.

### Mosquito maintenance and viral infections

A colony of *Culex pipiens f. quinquefasciatus* mosquitoes was maintained at 25°C and 65% humidity. Three to five day-old female mosquitoes (n = 60) were injected intrathoracically with 0.069 µl of dsRNAs against GFP, TRAF or Rel2. WNV (Kunjin strain; 1.13×10∧7 pfu/ml titer) or 199 medium (as control) was injected similarly 48 h later and the mosquitoes were incubated at 25°C and 65% humidity in an environmental cabinet (Thermoline Scientific, Smithfield, Australia) with a wet cotton pad (10% sucrose solution) provided daily as a food source. At 48 hpi, surviving females were collected for analysis. For each group, 2 females were homogenized using a disposable mortar-pestle and RNA extracted using the RNeasy kit (Qiagen) was used for real-time RT-qPCR as described above. The process was repeated again for creating duplicate samples.

### Statistical analysis

Standard error of the mean (sem) was calculated and data analyzed using the non-paired Student's *t*-test for single mean comparisons.

## Results

### Vago is activated by flavivirus infection in mosquito cells

**Figure 1:**
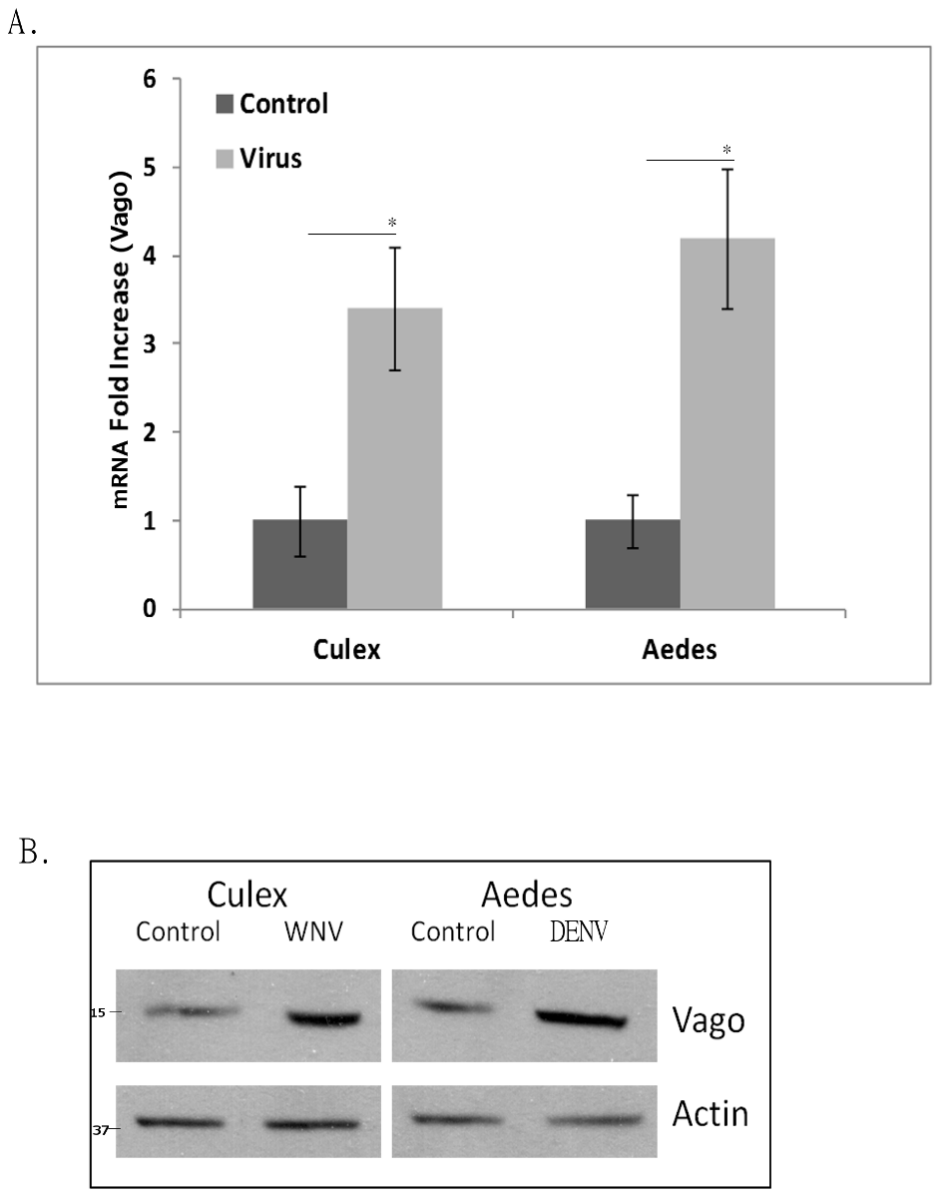
Vago is up-regulated by flavivirus infection in mosquito cells. (**A**) Hsu (*Culex quinquefasciatus*) and RML12 (*Aedes albopictus*) cells were infected with WNV and DENV, respectively, and total RNA was collected at 48 hpi. Real-time RT-qPCR was performed using Vago-specific primers. RpL32 (ribosomal protein L32) primers were used as an internal control. Error bars represents standard error from three separate experiments with assays performed in triplicate (Student's t-test *p<0.05, comparing mRNA level between control and virus-infected cells). (**B**) Cells treated as in (A) were lysed to harvest total protein samples. Western blotting was performed using anti-Vago and anti-beta actin antibodies.

*Culex* mosquitoes are known vectors of WNV and Japanese encephalitis virus, whilst *Aedes* mosquitoes are vectors of DENV and yellow fever virus. To determine whether Vago activation is conserved among flaviviruses and their vector species, *Culex quinquefasciatus* (Hsu) and *Aedes albopictus* (RML12) cell lines were infected with WNV and DENV, respectively, at a multiplicity of infection (MOI) of 5, and total RNA and protein lysates were collected at 48 h post-infection (hpi). Real-time RT-qPCR using primers specific for *Culex* (*Cx*) or *Aedes* (*Ae*) Vago mRNA indicated a 3–4 fold induction (Fig. 1A). Western blotting performed on protein lysates using anti-Vago antibody also showed a significant increase in Vago expression at 48 hpi (Fig. 1B). The results demonstrate that flavivirus-induced Vago activation is a conserved function in mosquitoes.

### Vago induction is dependent on NF-κB binding site in promoter

To identify the promoter region responsible for Vago activation, an initial analysis was conducted using PROMO software [24], which predicted multiple transcription factor binding regions in the 5′ region ∼2 kb upstream of *CxVago* gene (using *Culex quinquefasciatus* genome sequence, GI: 145648993) (Table S1). The 5′ regions ∼2 kb upstream from the transcription start site of *Vago* orthologs in *Culex quinquefasciatus*, *Aedes aegypti* and *Anopheles gambiae* mosquitoes were then aligned and conserved transcription factor binding sites were identified using phylogenetic footprinting (Footprinter2.0) [25]. The results indicated that NF-κB (Rel) (CACCTTCCCC) and GATA-1 (TTATCTTT) transcription factor binding sites are conserved in the Vago promoter region of the three mosquito species (Table S1).

**Figure 2:**
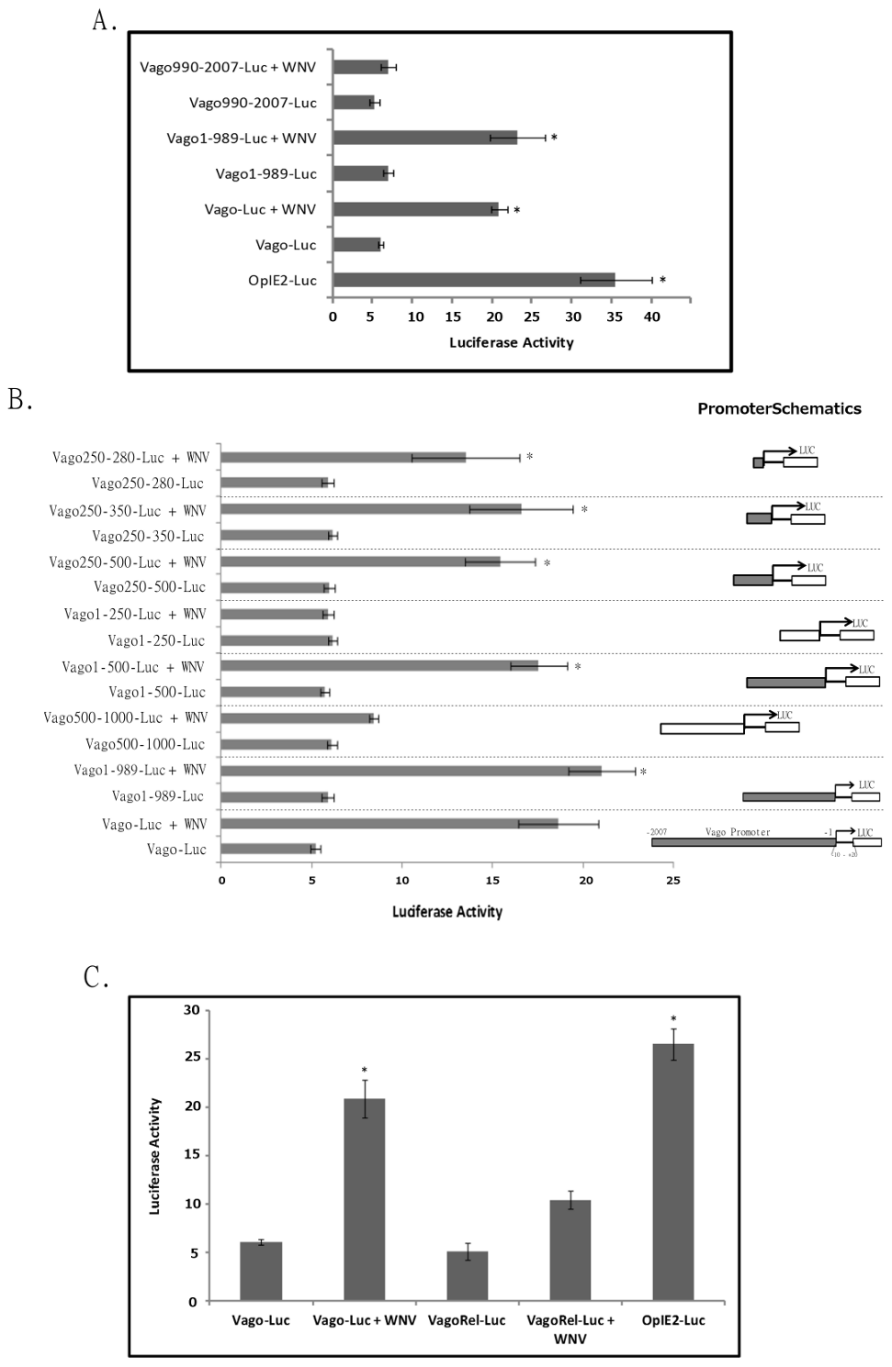
The Rel-binding site is responsible for WNV-induced *Cx*Vago activation. (**A**) Hsu cells were transfected with *Renilla* luciferase plasmid containing Vago promoter region (∼2 kb region upstream of gene, Vago-Luc) or ∼1 kb promoter fragments (Vago1-989-Luc and Vago990-2007-Luc). Cells were also transfected with firefly luciferase plasmid under the insect promoter (*OpIE2*) as an internal control. As a positive control, cells were transfected with Renilla luciferase under OpIE2 promoter (OpIE2-Luc). After 24 h, the cells were infected with WNV and luciferase activity was measured at 24 hpi. (**B**) Hsu cells were transfected with *Renilla* luciferase plasmid containing Vago promoter (Vago-Luc) or smaller fragments of the promoter along with plasmid containing the firefly luciferase reporter (OpIE2 promoter, internal control). After 24 h, the cells were infected with WNV and luciferase activity was measured at 24 hpi. The schematics of the promoter region used in the assay is provided in the right panel. The solid promoter regions were involved in inducing luciferase expression after WNV infection. (**C**) Hsu cells were transfected with *Renilla* luciferase plasmid containing Vago promoter (Vago-Luc) or promoter with a mutated Rel-binding site (VagoRel-Luc) along with firefly luciferase plasmid (OpIE2 promoter, internal control). As a positive control, cells were transfected with Renilla luciferase under OpIE2 promoter (OpIE2-Luc). After 24 h, the cells were infected with WNV and luciferase activity was measured at 24 hpi. The *Renilla* luciferase activity values were standardised using firefly luciferase activity values (R/F) and resulting values were plotted as bar graphs. Error bars represents standard error from three separate experiments with assays performed in triplicate and values were compared to ‘Vago-Luc’ (Student's t-test *p<0.05).

To validate the *in-silico* data identifying the promoter region responsible for Vago induction, a plasmid was constructed by inserting the ∼2 kb 5′ region upstream of the *CxVago* gene (−1 to −2007) in front of a luciferase reporter gene. Hsu cells transfected with this reporter plasmid were infected with WNV and luciferase activity in cell lysates was measured 24 hpi. The results indicated a significant (∼4-fold) increase in luciferase activity compared with uninfected control cells (Fig. 2A). Similarly, two fragments of the ∼2 kb promoter region (−1 to −989 and −990 to −2007) were cloned upstream of the luciferase reporter and transfected Hsu cells were infected with WNV. The results indicated a similar increase in luciferase activity in cells transfected with plasmid containing the −1 and −989 bp fragment (Fig. 2A), indicating this region is sufficient and essential for induction of Vago. As a control, Hsu cells transfected with a plasmid containing the whole promoter region (Vago-Luciferase) were treated with Vago-containing media, to determine whether Vago was acting in autocrine or paracrine fashion. The results showed no increase in luciferase activity indicating Vago is unable to activate its own promoter region (Fig. S3). The assay was then repeated using the luciferase reporter plasmid containing smaller fragments of the Vago promoter region of various lengths. The results indicated that a 30 bp region (−250 to −280) containing the predicted NF-κB binding site was sufficient to induce increased luciferase activity following WNV infection (Fig. 2B). To confirm that the NF-κB binding site is responsible for Vago induction, site-specific mutations (CTT to AGG) were introduced to disrupt the sequence (see Materials and Methods). WNV infection of Hsu cells transfected with the luciferase reporter plasmid containing mutated NF-κB binding site showed significantly less induction of luciferase activity compared with wild-type NF-κB binding site control (Fig. 2C). These results confirm that NF-κB binding site in the Vago promoter region is essential for its induction following WNV infection.

### Rel2 is required for Vago induction

**Figure 3:**
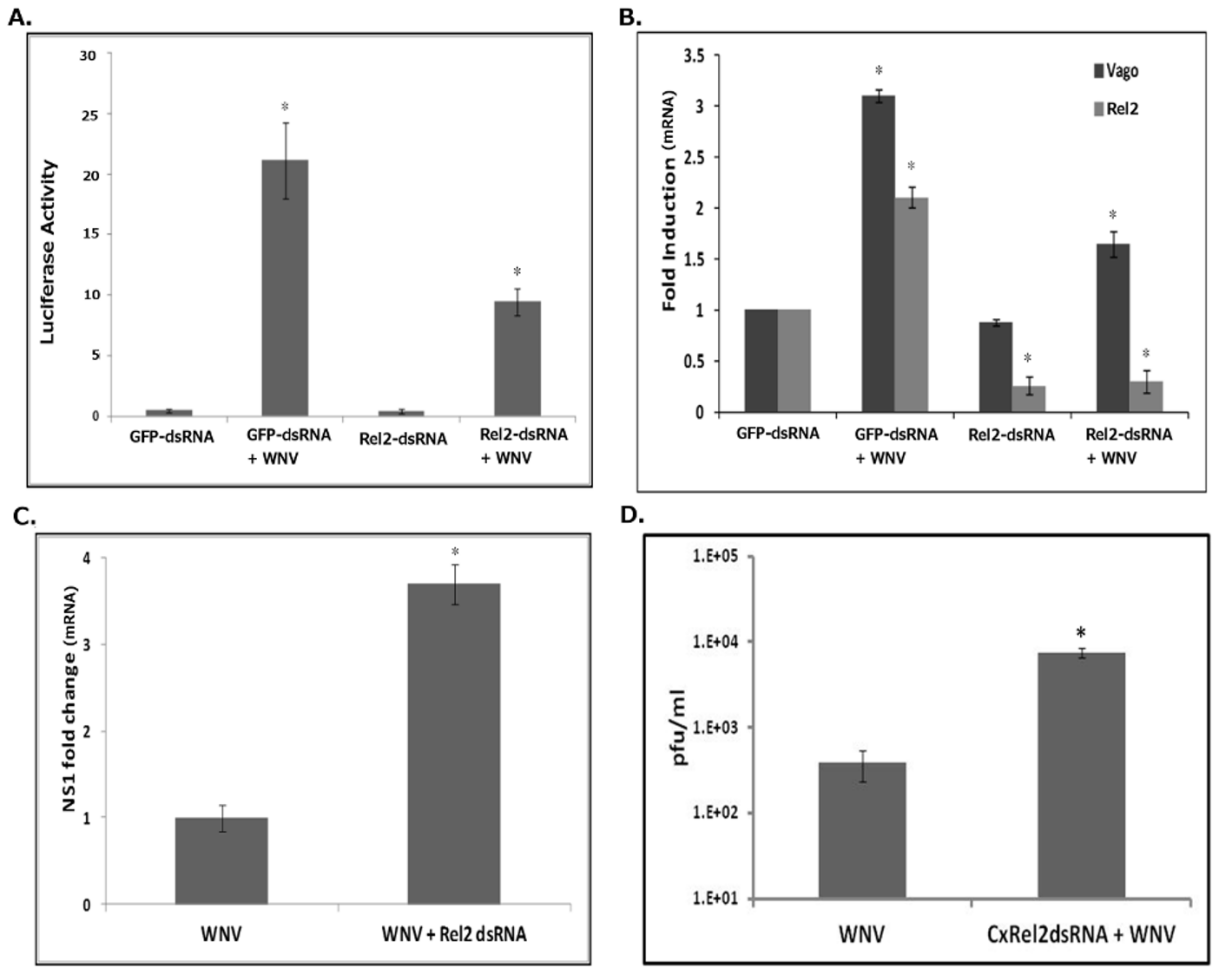
*Cx*Rel2 is required for activation of WNV-induced *Cx*Vago. (**A**) Hsu cells were transfected with dsRNA against Rel2 (Rel2-dsRNA) or control dsRNA (GFP-dsRNA) along with plasmid containing the Vago-Luciferase reporter (and firefly luciferase control plasmid). At 24 h post-transfection, the cells were infected with WNV and luciferase activity was measured at 24 hpi. The *Renilla* luciferase activity values were standardised using firefly luciferase activity values (R/F) and resulting values were plotted as bar graphs. * indicates p<0.05 compared to GFP-dsRNA. (**B–D**) Hsu cells were transfected with dsRNA against Rel2 (Rel2-dsRNA) or control dsRNA (GFP-dsRNA). At 24 h post-transfection, the cells were infected with WNV and total RNA was collected 48 hpi. (**B**) Real-time RT-qPCR was conducted using primers for *Cx*Vago, *Cx*Rel2 and *Cx*RpL32 (control). * indicates p<0.05 compared to GFP-dsRNA. (**C**) Real-time RT-qPCR was conducted using primers for WNV-NS1. * indicates p<0.05 compared with WNV infection. (**D**) Viral titer estimation by plaque assays conducted on the supernatant media from these cells at 48 hpi. Error bars represents standard error from three separate experiments with assays performed in triplicate (Student's t-test *p<0.05 compared to GFP-dsRNA).

Rel2 (XP_001862276) is the ortholog of human NF-κB (p105 subunit) in the *Culex* genome, with about 30% amino acid identity (Figure S1). Rel2 has previously been shown to be involved in the *Imd* pathway in mosquitoes [26], [27]. Experiments were performed to confirm the significance of Rel2 in Dicer-2-mediated induction of Vago. A long dsRNA was prepared to knockdown expression of Rel2 in *Culex* cells. Initially, Rel2 dsRNA and the luciferase reporter plasmid containing the −1 to −2007 Vago promoter region were co-transfected into Hsu cells. Cells co-transfected with GFP dsRNA were used as silencing control. The cells were then infected with WNV 24 h post-transfection and luciferase activity was assayed at 24 hpi. The results indicated a significant induction of luciferase activity in WNV-infected control cells treated with GFP dsRNA, but significantly less induction in WNV-infected cells treated with Rel2 dsRNA (Fig. 3A). The results show some induction of luciferase activity after WNV infection in cells transfected with Rel2 dsRNA. This may be due to inefficient knockdown of Rel2 or reduced transfection efficiency when cells are transfected with both Rel2 dsRNA and luciferase plasmids. We also cannot rule out other factors responsible for Vago regulation.

Next, to determine whether Vago induction requires activation of Rel2, Hsu cells were transfected with Rel2 dsRNA and then infected with WNV 24 h post-transfection. Control cells were transfected with GFP dsRNA. Total RNA and supernatant media were collected at 48 hpi. A real-time RT-qPCR assay on the total RNA using Rel2-specific primers showed that treatment with Rel2 dsRNA caused a significant decrease in Rel2 mRNA, indicating knockdown of the target (Fig. 3B). A real-time RT-qPCR using Vago-specific primers indicated that there was more than 3-fold induction of Vago mRNA after WNV infection but less than 2-fold increase in Vago mRNA in cells in which Rel2 was knocked-down, which although statistically significant, was much lower than in control cells (dsRNA GFP) infected with WNV (Fig. 3B). A western blot using Vago antibody also showed that Rel2 knockdown reduced Vago protein expression level after WNV infection (Figure S4). This suggested that Rel2 is required for Vago induction. A real-time qPCR assay was also performed using primers specific for WNV non-structural protein 1 (NS1) gene. The results indicated that viral RNA expression levels were >3 fold higher in the Rel2 knockdown cells than in control cells treated with GFP dsRNA (Fig. 3C). A plaque assay performed on the supernatant media showed significantly higher viral titers (>1 log) in Rel2-knockdown cells compared with control cells (Fig. 3D). The results indicate that Rel2 is required for induction of Vago and plays a significant role in antiviral immunity in mosquito cells.

### TRAF is essential for Vago induction after WNV infection

**Figure 4:**
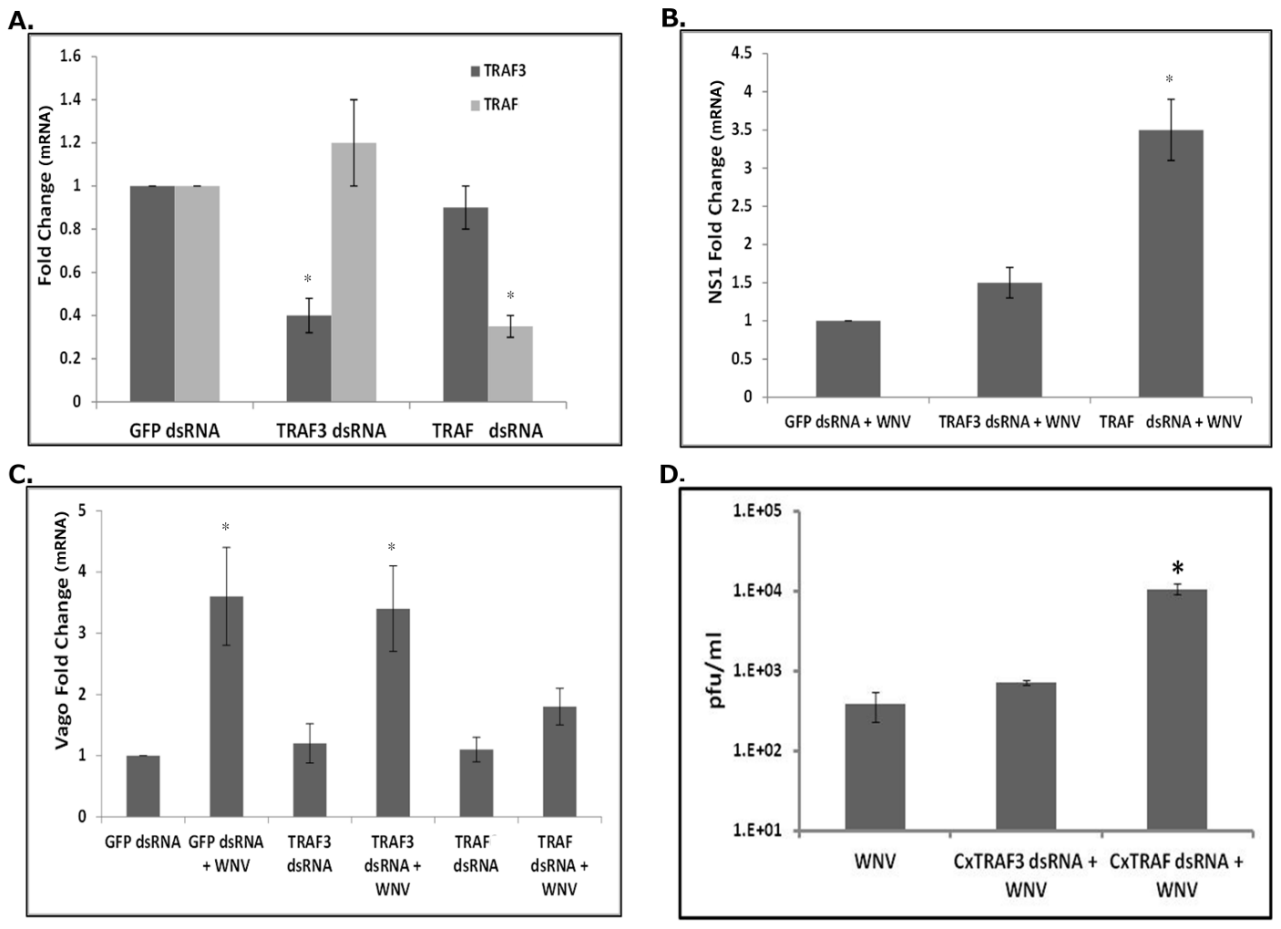
*Cx*TRAF, but not *Cx*TRAF3, is required for activation of WNV-induced *Cx*Vago. Hsu cells were transfected with dsRNA against TRAF-3 (TRAF-3 dsRNA) or TRAF (TRAF dsRNA). GFP dsRNA was used as a silencing control. At 24 h post-transfection, the cells were infected with WNV and total RNA was collected 48 hpi. (**A**) Real-time RT-qPCR was performed on uninfected cells using TRAF-3- or TRAF-specific primers. RpL32 primers were used as internal control. (**B**) Real-time RT-qPCR was performed using *Cx*Vago primers with RpL32 as control. (**C**) Real-time RT-qPCR was performed using WNV NS1 primers with RpL32 as control. (**D**) Viral titer estimation by plaque assays was conducted on the supernatant media from these cells at 48 hpi. Error bars represents standard error from three separate experiments with assays performed in triplicate (Student's t-test *p<0.05, compared to GFP dsRNA).

WNV infection in mammalian cells leads to activation of interferon regulatory factors (IRFs) and NF-κB via TRAF-3 and TRAF-6, respectively. Experiments were conducted to determine whether an equivalent pathway exists in *Culex* cells. The *Culex quinquefasciatus* genome encodes of two TRAF proteins, XP_001858793 (TRAF-3) and XP_001846690 (TRAF), with more than 30% amino acid similarity with human TRAF-3 and TRAF-6, respectively (Figure S2). Experiments were initially performed to confirm the significance of TRAFs in Dicer-2-mediated induction of Vago. Long dsRNAs were prepared to knockdown expression of each TRAF in *Culex* cells. Initially, TRAF3 or TRAF dsRNAs and the luciferase reporter plasmid containing the −1 to −2007 Vago promoter region were co-transfected into Hsu cells. Cells co-transfected with GFP dsRNA were used as silencing control. The cells were then infected with WNV 24 h post-transfection and luciferase activity was assayed at 24 hpi. The results indicated a significant induction of luciferase activity in WNV-infected control cells treated with GFP dsRNA, but significantly less induction in WNV-infected cells treated with TRAF dsRNA (Fig. S5). Hsu cells were then transfected with TRAF dsRNAs or GFP dsRNA (control) and infected with WNV at 24 h post-transfection. Total RNA and the supernatant media were collected at 48 hpi. Real-time RT-qPCR results indicated that TRAF-3 and TRAF dsRNA transfection led to significant silencing of the respective TRAF mRNA in cells without affecting expression of the other TRAF, indicating specificity and efficiency of gene silencing (Fig. 4A). A real-time RT-qPCR assay using *Culex* Vago primers showed that, compared to control cells, there was no significant WNV-induced increase in Vago mRNA in cells in which TRAF was silenced, suggesting that TRAF was required for Vago induction (Fig. 4C). However, Vago was upregulated in WNV-infected cells treated with TRAF-3 dsRNA, indicating TRAF-3 plays no role in Vago induction. A real-time RT-qPCR assay using WNV NS1 primers indicated that viral RNA levels were significantly higher (>3 fold) following TRAF knockdown compared to infected control cells treated with GFP dsRNA, whilst there was no significant difference in WNV RNA levels between infected cells with TRAF-3 dsRNA and control (GFP dsRNA) cells (Fig. 4B). Plaque assays performed on the supernatant media showed similar results with significantly higher viral titers (>1 log) in cells silenced for TRAF compared with control and TRAF-3 silenced cells (Fig. 4D). These results indicate that TRAF, but not TRAF-3, is essential for induction of Vago and in-turn plays a significant role in antiviral immunity in mosquito cells.

### WNV infection activates Rel2 in TRAF-dependent fashion

**Figure 5:**
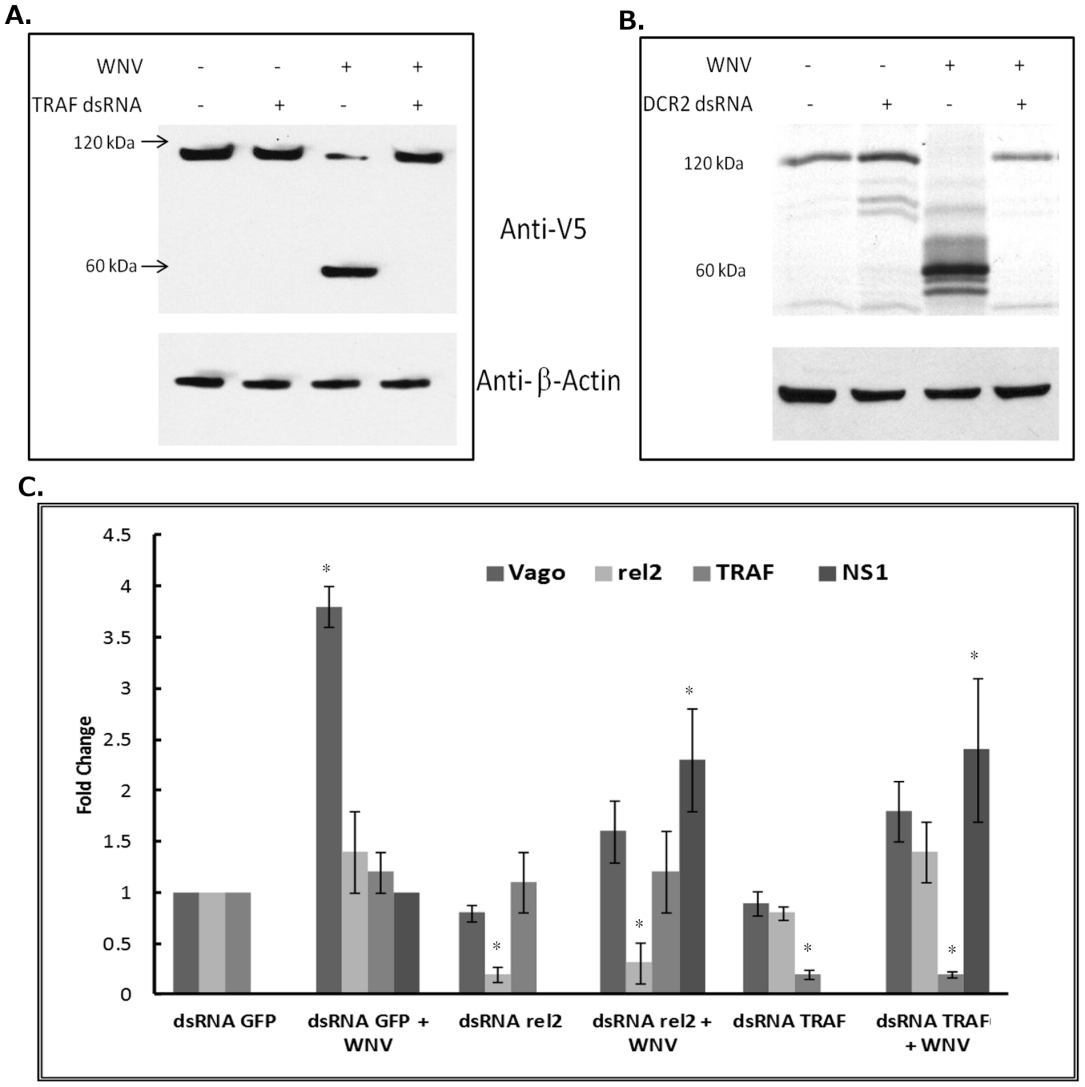
*Cx*Vago activation occurs via Dicer2-TRAF-Rel2 mediated pathway. Hsu cells were transfected with CxRel2 plasmid (with C-terminal V5 tag) and dsRNA against TRAF (**A**) or Dicer-2 (**B**). At 24 h post-transfection, the cells were infected with WNV and cell lysates was collected 48 hpi. Western blot was performed using anti-V5 and anti-beta actin antibodies. Representative blots shown here. (**C**) Female *Culex* mosquitoes were microinjected with dsRNAs against GFP, Rel2 or TRAF. After 48 h, the mosquitoes were infected with WNV (Kunjin strain). Total RNA was collected at 48 hpi and real-time RT-qPCR was performed using *Cx*Vago, *Cx*Rel2, *Cx*TRAF and WNV NS1 primers. Error bars represents standard error from experiment with assays performed in duplicates (Student's t-test *p<0.05 compared to dsRNA GFP).

Based on these results, it is possible to conclude that *Culex* Vago is activated by two different pathways, one involving TRAF and other involving Rel2. Therefore, experiments were conducted to determine whether both TRAF and Rel2 are part of the same Vago induction pathway downstream of Dicer-2. The *Culex* Rel2 gene was cloned into plasmid vector containing the insect cell promoter *OpIE*2 and a V5 tag (pIZ-V5). Hsu cells were then transfected with TRAF dsRNA (or GFP dsRNA as control) and the pIZ-Rel2-V5 plasmid. Cells were infected with WNV at 24 h post-transfection and cell lysates were collected at 48 hpi. Western blotting performed using anti-V5 antibody detected Rel2-V5 at ∼110 kDa (Fig. 5A). Cells infected with WNV showed cleavage of Rel2-V5 as evidenced by a band at ∼50 kDa, representing the carboxy-terminal inhibitory domain of the protein (IkB) tagged with V5. This indicated cleavage of Rel2 which is a prerequisite for nuclear localization of amino-terminal portion of Rel2 which acts as the activated transcription factor. Cells treated with TRAF dsRNA showed no evidence of cleavage of the ∼110 kDa Rel2-V5 band. This indicates that TRAF is required for WNV-induced activation of Rel2 in *Culex* cells. A similar experiment performed using Dicer-2 dsRNA indicated that Dicer-2 knockdown inhibited WNV-induced Rel2-V5 cleavage, compared with control cells (Fig. 5B). The results suggest that both Dicer-2 and TRAF are required for Rel2 activation.

### Rel2 and TRAF are required for Vago activation in Kunjin (WNV)-infected Culex mosquito

A mosquito infection model was used to validate results obtained *in vitro*. Adult 3–5 day old female mosquitoes (*Culex pipiens quinquefasciatus*) were microinjected with dsRNA against GFP, Rel2 or TRAF to knockdown specific genes. The mosquitoes were microinjected 48 h later with WNV (Kunjin strain) or cell culture medium (controls) and total RNA was collected at 48 hpi from a pool of 4 mosquitoes in each group. *Cx*Vago expression, as assayed by real-time RT-qPCR, was induced 3–4 fold in mosquitoes microinjected with GFP dsRNA and Kunjin virus. The results also showed that CxVago expression was not significantly induced in Kunjin challenged mosquitoes microinjected with Rel2 dsRNA or TRAF dsRNA, indicating involvement of these genes in WNV-induced Vago activation (Fig. 5C). The real-time RT-PCR results also showed specific knockdown of Rel2 and TRAF after dsRNA treatment, indicating efficient knockdown of the target genes (Fig. 5C). Changes in viral RNA were also measured by real-time RT-qPCR using WNV NS1 primers. The results indicated a statistically significant higher (>2fold) load of WNV RNA in the Rel2 and TRAF knockdown mosquitoes (Fig. 5C).

## Discussion

Mosquito antiviral immunity is a potentially important but poorly characterized aspect of arbovirus transmission. Mosquito immune response pathways could have a significant role in determining vector competence and in allowing efficient infection in the absence of significant pathology which is an intrinsic characteristic of the arbovirus transmission cycle in insects. The RNAi pathway is now known to comprise a major portion of mosquito defense against invading viruses [28]–[30]. Critically, this involves Dicer-2, an evolutionarily conserved protein containing a carboxy-terminal endoribonuclease domain which cleaves the viral RNA to form viral interfering RNAs. Recent research has also shown that the N-terminal helicase domain of Dicer-2 is similar to DExD/H-box helicase domain found in the mammalian viral RNA sensors RIG-I and MDA5 [16]. In response to viral infections, these mammalian proteins are involved in the induction of interferons which are subsequently secreted, stimulating antiviral activity in other cells. Our previous research has shown that *Culex* Vago is a functional homolog of mammalian interferons [15]. *Culex* Vago is induced by WNV infection and, like interferons, is secreted and activates Jak-STAT pathway in the neighboring cells, leading to induction of an antiviral response. However, neither the pathway linking Dicer-2 to Vago induction nor the antiviral effectors induced by Vago had previously been characterized.

The results presented here demonstrate that Vago induction occurs in response to flavivirus (WNV and DENV) infection in cells from a range of mosquito vector species and, using a WNV-*Culex* mosquito model, we reveal that this occurs through a novel immune signaling pathway. We show that detection of WNV infection by Dicer-2 leads to activation of TRAF, which in turn triggers cleavage of Rel2, allowing translocation of the N-terminal activation domain to the nucleus and binding to the NF-κB site in promoter region upstream of the *Vago* gene. As shown previously, induction and secretion of Vago then stimulates an antiviral response via the Jak-STAT pathway [15]. We also ruled out the possibility that Vago is activated by secretion of Vago from neighbouring cells (Figure S4). The Vago induction pathway is similar to the RIG-I/TRAF-6/NF-κB-mediated interferon activation pathway, which is activated in response to viral infections in mammalian cells. Interestingly, the mammalian pathway also involves the mitochondrial protein, MAVS, as well as interferon regulatory factors (IRFs), each of which have no apparent orthologs in the mosquito genome. Mammalian RIG-I contains a CARD domain which interacts with a CARD domain in MAVS [18]. This leads to activation of TRAF-3 which induces interferons in IRF-dependent pathway. Stimulation of MAVS also leads to activation of TRAF-6 which induces interferons in NF-κB-dependent fashion [31]. Unlike mammalian RIG-I, *Culex* Dicer-2 (and other Dicer-2 orthologs) lacks a CARD domain and seems to only involve TRAF and NF-κB dependent pathway. The mechanism of Dicer-2 interaction with the downstream proteins remains to be seen. The pathway may also involve unique and still unidentified proteins that link TRAF to Dicer-2. Experiments to identify these intermediates are currently underway. Using mutant flies and promoter deletion experiments, Deddouche et al [16] reported that induction of drosophila Vago was not mediated by members of NF-κB family of transcription factors. It is possible that regulation of Vago in mosquitoes is different than that in drosophila.

Interestingly, Rel2 is also activated by the Imd pathway in response to gram-negative bacterial infection in insects [32]. Peptidoglycans on gram-negative bacteria are recognized by PGRP receptor in insects, which leads to mutlimerization of the receptor. This receptor activation leads to recruitment of adaptor proteins, including Imd [33], which in turn leads to a cascading reaction involving kinases (TAK1 and IKK) which phospholyrate Rel2. Rel2 consists of an N-terminal nuclear factor containing domain (Rel-68) and an inhibitory C-terminal domain (Rel-49) which anchors Rel2 in the cytoplasm. Rel2 is then cleaved by the caspase, DREDD, releasing the N-terminal domain Rel-68. This translocates to the nucleus where it is able to activate transcription of genes encoding antimicrobial peptides [34]. In this report, we demonstrated that WNV-induced cleavage of Rel2 occurs via a distinct Dicer-2 and TRAF dependent pathway.

Overall, this report identifies a mechanism of activation of *Cx*Vago, an interferon functional homolog in mosquitoes. This Dicer-2-dependent pathway is similar to the process of activation of mammalian interferon and involves orthologs of TRAF and Rel2 (NF-κB). Although some of the intricacies of this pathway are yet to be established, the identification of this pathway opens up a novel avenue of mosquito antiviral response regulation. Furthermore, as Dicer-2 is an evolutionarily conserved protein with a canonical domain structure, it raises interesting questions about the evolutionary origins of innate antiviral immunity in vertebrates.

## Supporting Information

Figure S1 Protein sequence alignment of human NF-kB (p105 subunit; NP_003989) and Culex Rel2 (XP_001862276) using ClustalW2.
(DOCX)

Figure S2 Protein sequence alignment of Human (NP_665802) and Culex (XP_001846690) TRAF6 using ClustalW2.
(DOCX)

Figure S3 Hsu cells were transfected with plasmid containing the Vago-Luciferase (and firefly luciferase control plasmid). At 24 h post-transfection, the cells were infected with WNV or treated with Vago-containing media and luciferase activity was measured at 24 hpi. The *Renilla* luciferase activity values were standardised using firefly luciferase activity values (R/F) and resulting values were plotted as bar graphs. Error bars represents standard error from experiment with assays performed in triplicates (Student's t-test *p<0.05) and values were compared with control cells.
(DOCX)

Figure S4 Hsu cells were transfected with dsRNA against Rel2 (or GFP). At 24 h post-transfection, the cells were infected with WNV. At 48 hpi total cell lysates were collected and Western blot was performed using anti-Vago antibody. Anti-beta actin antibody was used as an internal loading control. Representative blot shown here.
(DOCX)

Figure S5 Hsu cells were transfected with dsRNA against TRAF (dsRNA TRAF), TRAF-3 (dsRNA TRAF3) or control dsRNA (GFP) along with plasmid containing the Vago-Luciferase reporter (and firefly luciferase control plasmid). At 24 h post-transfection, the cells were infected with WNV and luciferase activity was measured at 24 hpi. The *Renilla* luciferase activity values were standardised using firefly luciferase activity values (R/F) and resulting values were plotted as bar graphs. Error bars represents standard error from experiment with assays performed in triplicates (Student's t-test *p<0.05) and values were compared with GFP transfected cells.
(DOCX)

Table S1 Transcription factor binding sites on Culex Vago promoter region predicted by PROMO software. Footprinter2.0 was used to predict sites conserved between Culex, Aedes and Anopholes promoters. The conserved sites are in bold.
(DOCX)

Table S2 List of primers used in the manuscript for real-time PCR detection and dsRNA preparation.
(DOCX)
