# Supplementary material for: Dicer-2-Dependent Activation of Culex Vago Occurs via the TRAF-Rel2 Signaling Pathway
Source: PLoS Negl Trop Dis. 2014 Apr 24;8(4):e2823. doi: 10.1371/journal.pntd.0002823 (PMC3998923; doi:10.1371/journal.pntd.0002823)
Supplement: Figure S1 — Protein sequence alignment of human NF-kB (p105 subunit; NP_003989) and Culex Rel2 (XP_001862276) using ClustalW2. (DOCX) [file pntd.0002823.s001.docx]

Figure S1: Human NF-kB and Culex Rel2 protein sequence alignment by ClustalW2

NF-kB -------------------------MAEDDPYLGRPEQMFHLDPSLTHTIFNPEVFQPQM 35

Rel2 MEQHLQQPLSSSPTYSVLSMESSASSSSPSAVSTASSNMSPKSTTSETSSFNMQNLNISA 60

:. .. ..:* ..: : ** : :: .

NF-kB ALPTADG----------------------------PYLQILEQPKQRGFRFRYVCEGP-S 66

Rel2 SFPYYDDGSSSQMQTYHFTTGHGNAADHPQIELSVPHLVILEQPVDK-FRFRYQSEMHGT 119

::* *. *:* ***** :: ***** .* :

NF-kB HGGLPGASSEKNKKSYPQVKICNYVGPAKVIVQLVTNG-KNIHLHAHSLVGKHCEDGICT 125

Rel2 HGSLMGVHTEKSKKTFPSVELRGFQGEAKIRCSLFQVDPSKRAAHSHHLVIKSGEIDLID 179

**.* *. :**.**::*.*:: .: * **: .*. . .: *:* ** * * .:

NF-kB ----VTAGPKDMVVGFANLGILHVTKKKVFETLEARMTEACIRGYNPGLLVHPDLAYLQA 181

Rel2 PHDIEVNAETGYVAMFQGMGIIHTAKKNIAEELCKKIKRQRAVEMN-------------- 225

. . .. *. * .:**:*.:**:: * * ::.. *

NF-kB EGGGDRQLGDREKELIRQAALQQTKEMDLSVVRLMFTAFLPDST-GSFTRRLEPVVSDAI 240

Rel2 -----REISLREEHQLQKEAVEMAKTMNLNQVCLCFQAFQVDPVSGMWQQLCEPVYSNAI 280

*::. **:. ::: *:: :* *:*. * * * ** *.. * : : *** *:**

NF-kB YDSKAPNASNLKIVRMDRTAGCVTGGEEIYLLCDKVQKDDIQIRFYEEEE-NGGVWEGFG 299

Rel2 NNMKSALTGELKICRLSSTAGNIEGGEEVFMFVEKVCKNNIKIRFYELDEFDQEIWQDWG 340

: *:. :.:*** *:. *** : ****:::: :** *::*:***** :* : :*:.:*

NF-kB DFSPTDVHRQFAIVFKTPKYKDINITKPASVFVQLRRKSDLETSEPKPFLYYP--EIKDK 357

Rel2 TFSEADVHHQYAIAFKTPPYRNKDITEPAEVLMQLFRPRDKCQSEPVPFKYKPRPGMLAS 400

** :***:*:**.**** *:: :**:**.*::** * * *** ** * * : .

NF-kB EEVQRKRQKLMP-NFSDSFGGGSGAGAGGGGMFGSGGGGGGTGSTGPGYSFPHYGFPTYG 416

Rel2 ASSSRKRQRVHSGNISSEIPTTVPNDGGLAAMTAVAATAGPSRLPALHQPFPMVTQSTIS 460

. .****:: . *:*..: ..* ..* . .. .* : .. .** .* .

NF-kB GITFHPGTTKSNAGMKHG----------TMDTESKKDPEGCDKSDDKNTVNLFGKVIETT 466

Rel2 KEFNKSGIIQEILESNVQGHSGGPTGDITFNSGDFRDFIQCSSEDLHKLINEIGEAHEQS 520

:.* :. : *::: . :* *...* :: :* :*:. * :

NF-kB EQDQEPSEATVGNGEVTLTYATGTKEESAGVQDNLFLEKAMQLAKRHANALFDYAVTGDV 526

Rel2 KLETDAVASGSGPAGGSHQEAVRLERALESYLEANRHDKDVEILRKILAIIKLFGR--DY 578

: : :. : * . : *. :. . : :* ::: :: : :. *

NF-kB KMLLAVQRHLTAVQDENGDSVLHLAIIHLHSQLVRDLLEVTSGLISDDIINMRNDLYQTP 586

Rel2 ERCRELISALWMSSNKHKANCLHMAIERRESVISCKLVELLQEFQLHDLLGLVNDRNETA 638

: : * .::: . **:** : .* : .*:*: . : .*::.: ** :*.

NF-kB LHLAVITKQEDVVEDLLRAGADLSLLDRLGNSVLHLAAKEGHDKVLSILLKHKK-AALLL 645

Rel2 LHLAVFSNQVAVVESLLLAGVRISCCDYKGNSALHCAVVENCAESLDALVGHCKRNGLRW 698

*****:::* ***.** **. :* * ***.** *. *. : *. *: * * .*

NF-kB DHPNGDGLNAIHLAMMSNSLPCLLLLVAAGADVNAQEQKSGRTALHLAVEHDNISLAGCL 705

Rel2 DTANDDGYSPLQLAVMCRNLRVTKLLLDRGASPTERDLKHGNNILHIAVESDSLDLVNYI 758

* .*.** ..::**:*...* **: **. . :: * *.. **:*** *.:.*.. :

NF-kB LLEGDAHVDS-TTYDGTTPLHIAAGRGSTRLAALLKAAGADPLVENFEPLYDLDDSWENA 764

Rel2 LEQVDKSLSDEPNNAGYTPLQLANARHQVNASNKLIVR--ELLRFNPGGLLEKEPSNEDG 816

* : * :.. .. * ***::* .* ... : * . : * * * : : * *:.

NF-kB GEDEGVVPGTTPLDMATSWQVFDILNGKPYEPEFTSDDLLAQGDMKQLAEDVKLQLYKLL 824

Rel2 DEDD------------EEAATQDDLSAPPVEAGSSESVLLSMNLTCNRVEVIQLLENHVP 864

.**: . . * *.. * *. :.. **: . : .* ::* ::

NF-kB EIPDPDKNWATLAQKLGLGILNNAFRLSPAPSKTLMDNYEVSGGTVRELVEALRQMGYTE 884

Rel2 TAAELTP-LKTVPYGEGAGAVS---ELFDEPCLTELCELLNQNNVWKELGSLLDFNAFFS 920

.: *:. * * :. .* *. * : : .... :** . * .: .

NF-kB AIEVIQAASSPVKTTSQAHSLPLSPASTRQQIDELRDSDSVCDSGVETSFRKLSFTESLT 944

Rel2 IWEMSPNPAEMLLSYFEMQKMKLDHLIDILQALEQKEAIHCIDEMITTFMG-----IEAG 975

*: .:. : : : :.: *. * * ::: *. : * : .

NF-kB SGASLLTLNKMPHDYGQEGPLEGKI 969

Rel2 AKQQWMTYFRKPEGASSEGSMV--- 997

: . :* : *.. ..**.:
