# Supplementary material for: Dicer-2-Dependent Activation of Culex Vago Occurs via the TRAF-Rel2 Signaling Pathway
Source: PLoS Negl Trop Dis. 2014 Apr 24;8(4):e2823. doi: 10.1371/journal.pntd.0002823 (PMC3998923; doi:10.1371/journal.pntd.0002823)
Supplement: Figure S2 — Protein sequence alignment of Human (NP_665802) and Culex (XP_001846690) TRAF6 using ClustalW2. (DOCX) [file pntd.0002823.s002.docx]

Figure S2: Human and Culex TRAF6 protein sequence alignment by ClustalW2

HsTRAF6 MSLLNCENSCGSSQSESDCCVAMASSCSAVTKDDSVGGTASTGNLSSSFMEEIQGYDVEF 60

CxTRAF6 ----------------------MVRSLSQWTKTLSFPARISPNRNSKDCSVNVLPITPPP 38

*. * * ** *. . *... *.. ::

HsTRAF6 DPP-LESKYECPICLMALREAVQTPCGHRFCKACIIKSIRDAGHKCPVDNEILLENQLFP 119

CxTRAF6 APPRNKPATSTTTCSSSNSSTMSSPSPPPNVPITEISQIVTP-------ILSLLPFQIYP 91

** :. . . * : .::.:*. *..* . ** *::*

HsTRAF6 DNFAKREILSLMVKCPN--EGCLHKMELRHLEDHQAHCEFALMDCP-QCQRPFQKFHINI 176

CxTRAF6 DPESEKAIMGSLVFCIHHKQGCKWSDELRKLKAHLNTCKHDAIPCPNKCGSQIPRVMMTD 151

* ::: *:. :* * : :** . ***:*: * *:. : ** :* : :. :.

HsTRAF6 HILKDCPRRQVSCDNCAASMAFEDKEIHDQNCPLANVICEY-CNTILIREQMPNHYDLDC 235

CxTRAF6 HLAFTCILRRAICEFCNVEFTGLGLEEHAGTCSSEPMYCESKCGARVVRGRMSIHRAKDC 211

*: * *:. *: * ..:: . * * .*. : ** *.: ::* :*. * **

HsTRAF6 PTAPIPCTFSTFGCHEKMQRNHLARHLQENTQSHMR----MLAQAVHSLSVIPDSGYISE 291

CxTRAF6 SKRLRRCPHCSREFSADTLSAHGATCPRSPVPCPQRCDAGPMARADLDSHLRDECKALSV 271

.. *...: . * * :. . . * :*:* . : :. :*

HsTRAF6 VRNFQETIHQLEG-RLVRQDHQIRELTAKMETQSMYVSELKRTIRTLEDKVAEIEAQQCN 350

CxTRAF6 PCSFKDAGCRFKGPRHLLEAHLESNTSAHLSLMVALSGRQGQQITMLKNAMAKLSTN-YT 330

.*::: :::* * : : * : :*::. .. : * *:: :*::.:: .

HsTRAF6 GIYIWKIGNFGMHLKCQEEEKPVVIHSPGFYTGKPGYKLCMRLHLQLPTAQRCANYISLF 410

CxTRAF6 GTLLWKITDWSAKMVEAKSKDGLELVSPPFYTSQYGYKLQASMFLNG-NGPGESTHVSVY 389

* :*** ::. :: :.:. : : ** ***.: **** :.*: .. :.::*::

HsTRAF6 VHTMQGEYDSHLPWPFQGTIRLTILDQSEAPVRQNHEEIMDAKPELLAFQRPTIPRNPKG 470

CxTRAF6 IKVLPGEYDALLKWPFSHSVTFTLFEQGTLGGQGGVAESFVPDPSWENFQRPSSEPDALG 449

::.: ****: * ***. :: :*:::*. : . * : ..*. ****: :. *

HsTRAF6 FGYVTFMHLEALRQRTFIKDDTLLVRCEVSTRFDMGSLRREGFQPRSTDAGV 522

CxTRAF6 FGFPRFVSHELLNRRPFVREDTVFLRVKVDPSKIVAV--------------- 486

**: *: * *.:*.*:::**:::* :*.. :.
