# Supplementary material for: Dicer-2-Dependent Activation of Culex Vago Occurs via the TRAF-Rel2 Signaling Pathway
Source: PLoS Negl Trop Dis. 2014 Apr 24;8(4):e2823. doi: 10.1371/journal.pntd.0002823 (PMC3998923; doi:10.1371/journal.pntd.0002823)
Supplement: Figure S3 — Hsu cells were transfected with plasmid containing the Vago-Luciferase (and firefly luciferase control plasmid). At 24 h post-transfection, the cells were infected with WNV or treated with Vago-containing media and luciferase activity was measured at 24 hpi. The Renilla luciferase activity values were standardised using firefly luciferase activity values (R/F) and resulting values were plotted as bar graphs. Error bars represents standard error from experiment with assays performed in triplicates (Student's t-test *p<0.05) and values were compared with control cells. (DOCX) [file pntd.0002823.s003.docx]

Figure S3: Vago treatment does not activate luciferase reporter activity.
