# Supplementary material for: Dicer-2-Dependent Activation of Culex Vago Occurs via the TRAF-Rel2 Signaling Pathway
Source: PLoS Negl Trop Dis. 2014 Apr 24;8(4):e2823. doi: 10.1371/journal.pntd.0002823 (PMC3998923; doi:10.1371/journal.pntd.0002823)
Supplement: Figure S4 — Hsu cells were transfected with dsRNA against Rel2 (or GFP). At 24 h post-transfection, the cells were infected with WNV. At 48 hpi total cell lysates were collected and Western blot was performed using anti-Vago antibody. Anti-beta actin antibody was used as an internal loading control. Representative blot shown here. (DOCX) [file pntd.0002823.s004.docx]

Figure S4: Rel2 is required for Vago induction after WNV infection
