# Supplementary material for: Dicer-2-Dependent Activation of Culex Vago Occurs via the TRAF-Rel2 Signaling Pathway
Source: PLoS Negl Trop Dis. 2014 Apr 24;8(4):e2823. doi: 10.1371/journal.pntd.0002823 (PMC3998923; doi:10.1371/journal.pntd.0002823)
Supplement: Figure S5 — Hsu cells were transfected with dsRNA against TRAF (dsRNA TRAF), TRAF-3 (dsRNA TRAF3) or control dsRNA (GFP) along with plasmid containing the Vago-Luciferase reporter (and firefly luciferase control plasmid). At 24 h post-transfection, the cells were infected with WNV and luciferase activity was measured at 24 hpi. The Renilla luciferase activity values were standardised using firefly luciferase activity values (R/F) and resulting values were plotted as bar graphs. Error bars represents standard error from experiment with assays performed in triplicates (Student's t-test *p<0.05) and values were compared with GFP transfected cells. (DOCX) [file pntd.0002823.s005.docx]

Figure S5: *Cx*TRAF is required for activation of WNV-induced *Cx*Vago
