# Supplementary material for: Dicer-2-Dependent Activation of Culex Vago Occurs via the TRAF-Rel2 Signaling Pathway
Source: PLoS Negl Trop Dis. 2014 Apr 24;8(4):e2823. doi: 10.1371/journal.pntd.0002823 (PMC3998923; doi:10.1371/journal.pntd.0002823)
Supplement: Table S1 — Transcription factor binding sites on Culex Vago promoter region predicted by PROMO software. Footprinter2.0 was used to predict sites conserved between Culex, Aedes and Anopholes promoters. The conserved sites are in bold. (DOCX) [file pntd.0002823.s006.docx]

**Supplementary Table 1:**

Transcription factor list from PROMO:

| \| **Factor name** \| **Start position** \| **End position** \| \| --- \| --- \| --- \| \| C/EBPbeta [T00017] \| -2014 \| -2007 \| \| C/EBPbeta [T00017] \| -1204 \| -1197 \| \| C/EBPbeta [T00017] \| -908 \| -901 \| \| C/EBPbeta [T00017] \| -588 \| -581 \| \| C/EBPbeta [T00017] \| -388 \| -381 \| \| C/EBPbeta [T00017] \| -18 \| -11 \| \| USF-1 [T00875] \| -1659 \| -1653 \| \| USF-1 [T00875] \| -1526 \| -1520 \| \| USF-1 [T00875] \| -472 \| -466 \| \| USF-1 [T00875] \| -448 \| -442 \| \| USF-1 [T00875] \| -358 \| -352 \| \| Pax-2 [T01823] \| -1967 \| -1961 \| \| Pax-2 [T01823] \| -728 \| -722 \| \| Pax-2 [T01823] \| -642 \| -636 \| \| Pax-2 [T01823] \| -500 \| -494 \| \| Pax-8 [T01828] \| -1966 \| -1961 \| \| Pax-8 [T01828] \| -1685 \| -1680 \| \| Pax-8 [T01828] \| -1279 \| -1274 \| \| Pax-8 [T01828] \| -1197 \| -1192 \| \| Pax-8 [T01828] \| -1190 \| -1185 \| \| Pax-8 [T01828] \| -1131 \| -1126 \| \| Pax-8 [T01828] \| -1047 \| -1042 \| \| Pax-8 [T01828] \| -978 \| -973 \| \| Pax-8 [T01828] \| -370 \| -365 \| \| Pax-8 [T01828] \| -41 \| -36 \| \| c-Jun [T00131] \| -1969 \| -1964 \| \| c-Jun [T00131] \| -1798 \| -1793 \| \| c-Jun [T00131] \| -1765 \| -1760 \| \| c-Jun [T00131] \| -1636 \| -1631 \| \| c-Jun [T00131] \| -893 \| -888 \| \| c-Jun [T00131] \| -725 \| -720 \| \| c-Jun [T00131] \| -484 \| -479 \| \| GATA-1 [T00305] \| -1905 \| -1898 \| \| GATA-1 [T00305] \| -598 \| -591 \| \| **GATA-1 [T00305]** \| -171 \| -164 \| \| TFIIB [T00818] \| -1897 \| -1890 \| \| TFIIB [T00818] \| -1366 \| -1359 \| \| TFIIB [T00818] \| -1345 \| -1338 \| \| TFIIB [T00818] \| -1045 \| -1038 \| \| TFIIB [T00818] \| -899 \| -892 \| \| TFIIB [T00818] \| -664 \| -657 \| \| TBP [T00798] \| -1844 \| -1837 \| \| TBP [T00798] \| -1186 \| -1179 \| \| TBP [T00798] \| -816 \| -809 \| \| TBP [T00798] \| -427 \| -420 \| \| TBP [T00798] \| -165 \| -158 \| \| Vpr [T02399] \| -1762 \| -1755 \| \| Vpr [T02399] \| -1503 \| -1496 \| \| Vpr [T02399] \| -460 \| -453 \| \| NF-1 [T00538] \| -1508 \| -1501 \| \| NF-1 [T00538] \| -1488 \| -1481 \| \| NF-1 [T00538] \| -1087 \| -1080 \| \| NF-1 [T00538] \| -1056 \| -1049 \| \| NF-1 [T00538] \| -1005 \| -998 \| \| NF-1 [T00538] \| -869 \| -862 \| \| NF-1 [T00538] \| -322 \| -315 \| \| Sox2 [T01836] \| -1978 \| -1972 \| \| Sox2 [T01836] \| -1697 \| -1691 \| \| Sox2 [T01836] \| -1418 \| -1412 \| \| Sox2 [T01836] \| -1266 \| -1260 \| \| Sox2 [T01836] \| -992 \| -986 \| \| Sox2 [T01836] \| -725 \| -719 \| \| Sox2 [T01836] \| -304 \| -298 \| \| Sox2 [T01836] \| -65 \| -59 \| \| TFIID [T00820] \| -1742 \| -1735 \| \| TFIID [T00820] \| -1190 \| -1183 \| \| TFIID [T00820] \| -1184 \| -1177 \| \| TFIID [T00820] \| -408 \| -401 \| \| Antp [T00026] \| -187 \| -182 \| \| Antp [T00026] \| -183 \| -178 \| \| Antp [T00026] \| -105 \| -100 \| \| Antp [T00026] \| -73 \| -68 \| \| STAT1beta [T01573] \| -1834 \| -1825 \| \| STAT1beta [T01573] \| -1357 \| -1348 \| \| STAT1beta [T01573] \| -362 \| -353 \| \| TBP [T00794] \| -412 \| -405 \| \| TBP [T00794] \| -408 \| -401 \| \| FOXN2 [T04206] \| -1466 \| -1456 \| \| HNF-1 [T00369] \| -1387 \| -1376 \| \| C/EBPalpha [T00104] \| -1551 \| -1546 \| \| C/EBPalpha [T00104] \| -97 \| -92 \| \| C/EBPalpha [T00104] \| -14 \| -9 \| \| Hlf [T01071] \| -962 \| -954 \| \| Hlf [T01071] \| -440 \| -432 \| \| Hlf [T01071] \| -287 \| -279 \| \| HBP-1 [T00354] \| -936 \| -929 \| \| NF-Y [T00150] \| -1647 \| -1640 \| \| FOXO4 [T04176] \| -521 \| -512 \| \| FOXO4 [T04176] \| -105 \| -96 \| \| AP-1 [T00031] \| -501 \| -491 \| \| **Rel [T00594]** \| -267 \| -258 \| \| STAT6 [T01581] \| -263 \| -255 \| \| FOXO1 [T04203] \| -283 \| -273 \| \| FOXO1 [T04203] \| -239 \| -229 \| \| FOXO1 [T04203] \| -201 \| -191 \| \| c-Ets-1 [T00111] \| -1599 \| -1590 \| \| c-Ets-1 [T00111] \| -266 \| -257 \| \| USF-1 [T00877] \| -627 \| -617 \| \| TFII-I [T00824] \| -1771 \| -1761 \| \| Smad3 [T04096] \| -2005 \| -1996 \| \| Smad4 [T04292] \| -2006 \| -1996 \| \|  \|  \|  \| |
| --- | --- | --- | --- | --- | --- | --- | --- | --- | --- | --- | --- | --- | --- | --- | --- | --- | --- | --- | --- | --- | --- | --- | --- | --- | --- | --- | --- | --- | --- | --- | --- | --- | --- | --- | --- | --- | --- | --- | --- | --- | --- | --- | --- | --- | --- | --- | --- | --- | --- | --- | --- | --- | --- | --- | --- | --- | --- | --- | --- | --- | --- | --- | --- | --- | --- | --- | --- | --- | --- | --- | --- | --- | --- | --- | --- | --- | --- | --- | --- | --- | --- | --- | --- | --- | --- | --- | --- | --- | --- | --- | --- | --- | --- | --- | --- | --- | --- | --- | --- | --- | --- | --- | --- | --- | --- | --- | --- | --- | --- | --- | --- | --- | --- | --- | --- | --- | --- | --- | --- | --- | --- | --- | --- | --- | --- | --- | --- | --- | --- | --- | --- | --- | --- | --- | --- | --- | --- | --- | --- | --- | --- | --- | --- | --- | --- | --- | --- | --- | --- | --- | --- | --- | --- | --- | --- | --- | --- | --- | --- | --- | --- | --- | --- | --- | --- | --- | --- | --- | --- | --- | --- | --- | --- | --- | --- | --- | --- | --- | --- | --- | --- | --- | --- | --- | --- | --- | --- | --- | --- | --- | --- | --- | --- | --- | --- | --- | --- | --- | --- | --- | --- | --- | --- | --- | --- | --- | --- | --- | --- | --- | --- | --- | --- | --- | --- | --- | --- | --- | --- | --- | --- | --- | --- | --- | --- | --- | --- | --- | --- | --- | --- | --- | --- | --- | --- | --- | --- | --- | --- | --- | --- | --- | --- | --- | --- | --- | --- | --- | --- | --- | --- | --- | --- | --- | --- | --- | --- | --- | --- | --- | --- | --- | --- | --- | --- | --- | --- | --- | --- | --- | --- | --- | --- | --- | --- | --- | --- | --- | --- | --- | --- | --- | --- | --- | --- | --- | --- | --- | --- | --- | --- | --- | --- | --- | --- | --- | --- | --- | --- | --- | --- | --- | --- | --- | --- | --- | --- | --- | --- |
