# Supplementary material for: Dicer-2-Dependent Activation of Culex Vago Occurs via the TRAF-Rel2 Signaling Pathway
Source: PLoS Negl Trop Dis. 2014 Apr 24;8(4):e2823. doi: 10.1371/journal.pntd.0002823 (PMC3998923; doi:10.1371/journal.pntd.0002823)
Supplement: Table S2 — List of primers used in the manuscript for real-time PCR detection and dsRNA preparation. (DOCX) [file pntd.0002823.s007.docx]

Supplementary Table 2:

Primers used in the manuscript for detection or dsRNA preparation

| **Primer Name** | | **Sequence** |
| --- | --- | --- |
| **CxVago-Forward** | | CCC TGG AGT CGG CGA AAC TGC |
| **CxVago-Reverse** | | CAC GAG CAC GGA ACC GCA AGT |
| **AeVago-Forward** | | TGA CGA GGA GAA ATC CAT CC |
| **AeVago-Reverse** | | TCC CAT CGT GTA CGC ATT TA |
| **VagoPromoter-Forward** | | CGGATGTGTGTAGGTGGGTG |
| **VagoPromoter-Reverse** | | AGACTGAGTTCGCTGGGTCA |
| **WNV-NS1-Forward** | | ATCGCGCTTGGAATAGCTTA |
| **WNV-NS1-Reverse** | | GACAGCCGTTCCAATGATCT |
| **GFP-dsRNA-Forward** | | GAATTAATACGACTCACTATAGGGAGATGTCACTACTTTCACTTATG |
| **GFP-dsRNA-Reverse** | | GAATTAATACGACTCACTATAGGGAGAGAACGCTTCCATCTTCAATG |
| **CxRpL32-Forward** | CGA GCA GCA GTT GCC CAG CT | |
| **CxRpL32-Reverse** | GCT GAA GGG GTC CGG GTT GC | |
| **CxRel2-Forward** | | AGTGGACAAGTCACTCAGCG |
| **CxRel2-Reverse** | | TCAGTTTGTTGCTCGCGTTG |
| **CxRel2dsRNA-Forward** | | GAATTAATACGACTCACTATAGGGAGATGAGCGGGATGTGGCAGCAG |
| **CxRel2dsRNA-Reverse** | | GAATTAATACGACTCACTATAGGGAGACTGCTGCCACATCCCGCTCA |
| **CxRel2Clon-Forward** | | ATGGAGCAACATCTGCAGCA |
| **CxRel2Clon-Reverse** | | TCAGACCATGCTTCCTTCGG |
| **CxTRAF3-Forward** | | ATAGGCCGTTTGGGTACACG |
| **CxTRAF3-Reverse** | | TACCCGTTGACGACCACAAG |
| **CxTRAF3dsRNA-Forward** | | GAATTAATACGACTCACTATAGGGAGAGAAAAGTACCGCCGCGAAAC |
| **CxTRAF3dsRNA-Reverse** | | GAATTAATACGACTCACTATAGGGAGATCACCGTTTTGGAAAAATCC |
| **CxTRAF6-Forward** | | CTGCAAGCATGATGCGATCC |
| **CxTRAF6-Reverse** | | TTGCAAAACTCGCAGATGGC |
| **CxTRAF6dsRNA-Forward** | | GAATTAATACGACTCACTATAGGGAGAACCTGCAAGCATGATGCGAT |
| **CxTRAF6dsRNA-Reverse** | | GAATTAATACGACTCACTATAGGGAGAATCTGCCCGAGCCATCGGAC |
